# The Essentials of *PgPG1*, a Polygalacturonase-Encoding Gene for the Invasion of *Pyrenophora graminea* to *Hordeum vulgare*

**DOI:** 10.3390/ijms26062401

**Published:** 2025-03-07

**Authors:** Erjing Si, Ming Guo, Haiying Liu, Chengdao Li, Juncheng Wang, Lirong Yao, Yaxiong Meng, Xiaole Ma, Baochun Li, Ke Yang, Xunwu Shang, Huajun Wang

**Affiliations:** 1 State Key Laboratory of Aridland Crop Science, Gansu Key Laboratory of Crop Improvement and Germplasm Enhancement, Lanzhou 730070, China; sierjing@163.com (E.S.); 18794807552@163.com (M.G.); 19527377701@163.com (H.L.); wangjc@gsau.edu.cn (J.W.); ylr0384@163.com (L.Y.); yxmeng1@163.com (Y.M.); maxl@gsau.edu.cn (X.M.); libc@gsau.edu.cn (B.L.); yk_831116@163.com (K.Y.); 17393294106@163.com (X.S.); 2 College of Agronomy, Gansu Agricultural University, Lanzhou 730070, China; 3 Western Barley Genetics Alliance, College of Science, Health, Engineering and Education, Murdoch University, Murdoch, WA 6150, Australia; c.li@murdoch.edu.au; 4 College of Life Science and Technology, Gansu Agricultural University, Lanzhou 730070, China

**Keywords:** *Pyrenophora graminea*, polygalacturonase, gene family, pathogenicity

## Abstract

Barley leaf stripe, caused by *Pyrenophora graminea*, significantly reduces yield. Polygalacturonase, a key fungal pectinase, facilitates cell wall degradation for nutrition acquisition and colonization. To determine whether *P. graminea* contains polygalacturonase (*PgPG*)-encoding genes and their role in pathogenicity, four *PgPG* genes (*PgPG1*–*PgPG4*) were identified in the *P. graminea* genome. Quantitative RT-PCR revealed that *PgPG1* had the highest inducible expression during barley infection, suggesting its critical vital role in pathogenesis. *PgPG1* was silenced and overexpressed in *P. graminea* QWC (wild-type) using CaCl_2_-PEG4000-mediated protoplast transformation. The *PgPG1* RNAi mutants exhibited slower growth, while overexpression mutants grew faster. Relative to the wild-type, the disease incidence of Alexis, a highly susceptible barley variety, decreased by 62.94%, 42.19%, 45.74%, and 40.67% for RNAi mutants, and increased by 12.73%, 12.10%, 12.63%, and 10.31% for overexpression mutants. Pathogenicity analysis showed decreased disease incidence with *PgPG1* RNAi mutants and increased severity with overexpression mutants. Trypan blue staining and polygalacturonase activity assays confirmed that overexpression mutants caused more severe damage compared to wild-type and RNAi mutants. These findings indicate that *PgPG1* plays a vital role in the pathogenicity of *P. graminea* in barley and has great potential as a pathogen target gene to develop a durable resistance variety to *P. graminea*.

## 1. Introduction

Barley leaf stripe, caused by *Pyrenophora graminea*, is widespread in many countries and regions, including Europe, North America, North Africa, Russia, India, and China [1]. A 1% increase in disease incidence reduces production by 1% [2]; when the disease incidence is high, it leads to significant yield and economic losses, underscoring the need for effective disease control strategies [3,4]. Despite its importance, the pathogenic mechanisms of *P. graminea* remain poorly understood, with only the mitogen-activated protein kinase kinase gene (*PGPBS*) identified as contributing to *P. graminea* pathogenicity [5]. Research has shown that *P. graminea* mycelia invade the host by spreading within the middle lamella during infection [6,7]. Since the middle lamella primarily comprises pectin, its degradation by *P. graminea*-secreted enzymes is likely critical for colonization. Among the key enzymes involved in pectin degradation are polygalacturonase (PG), pectate lyase, and pectin methyl esterase. Polygalacturonases break down pectin in plant cell walls, facilitating nutrient release to support pathogen growth and development [8,9,10,11,12].

Molecular genetic studies on other plant pathogens have highlighted the role of PG-encoding genes in fungal pathogenicity. For instance, polygalacturonase P2c in *Aspergillus flavus* influences virulence by enhancing host invasion, as demonstrated through gene expression and targeted disruption of the *pecA* gene [13]. Similarly, the *Bcpg1* gene in *Botrytis cinerea* and the *PG1* gene in *Fusarium oxysporum* contributed to host infection [14]. Targeted disruption of PG-encoding genes in other fungi, such as *Alternaria citri* [15], *Claviceps purpurea* [16], and *Xylella fastidiosa* [17], has also resulted in weakened or lost pathogenicity, further underscoring the importance of PGs in fungal virulence.

However, not all *PG* genes are essential for pathogenicity. For instance, disrupting the *enPG1* in *Cryphonectria parasitica* [18] or the *PG5* gene in *F. oxysporum* [19] had no measurable effect on pathogenicity, likely due to functional redundancy among PG isozymes. Similarly, the *AaPG1* gene in *A. alternata* did not impact virulence, as mutants exhibited the same disease symptoms as the wild-type [15]. These findings suggest that while PGs are critical for the virulence of many plant pathogens, their contribution can vary depending on the organism and host–pathogen interaction.

Little is known about PG-encoding genes in *P. graminea*. All known PG genes belong to the glycosylhydrolase gene family (GH28), which hydrolyzes glycosidic bonds. RNA interference (RNAi) technology based on the *pSilent-1* vector has been successfully applied in *P. graminea* [5], offering a valuable tool for functional gene studies in this pathogen. Polygalacturonase production is critical for the success and survival of many fungal pathogens during host infection [20]. However, the specific contribution of PGs to *P. graminea* pathogenicity in barley remains unclear. To address this, we identified members of the GH28 gene family in *P. graminea* and selected *PgPG1* as a key candidate gene. We investigated its subcellular localization, validated its signal peptide secretion function, and evaluated its role in pathogenicity by comparing the virulence of RNAi and overexpression mutants with that of the wild-type strain QWC. This study will provide the pathogen target gene for the creation of disease-resistant germplasm based on host-induced gene silencing.

## 2. Results

### 2.1. Identification of PgPG Proteins in P. graminea

Four *Pg* genes, designated *PgPG1*–*PgPG4*, were identified at the genomic level (Table 1). These genes were predicted to encode polygalacturonase enzymes, suggesting a potential role in pectin degradation in barley. Sequence analysis using the InterPro database confirmed their involvement in carbohydrate metabolism. The proteins encoded by these genes ranged from 370 to 705 aa, with molecular weights between 37.04 and 74.98 kD. Signal peptides were detected in all four proteins (Figure S2), and their subcellular localization was predicted to be extracellular, identifying them as apoplastic proteins (Table 1).

### 2.2. Gene Structure and Conserved Motif Analysis of PgPG Proteins

**Figure 1:**
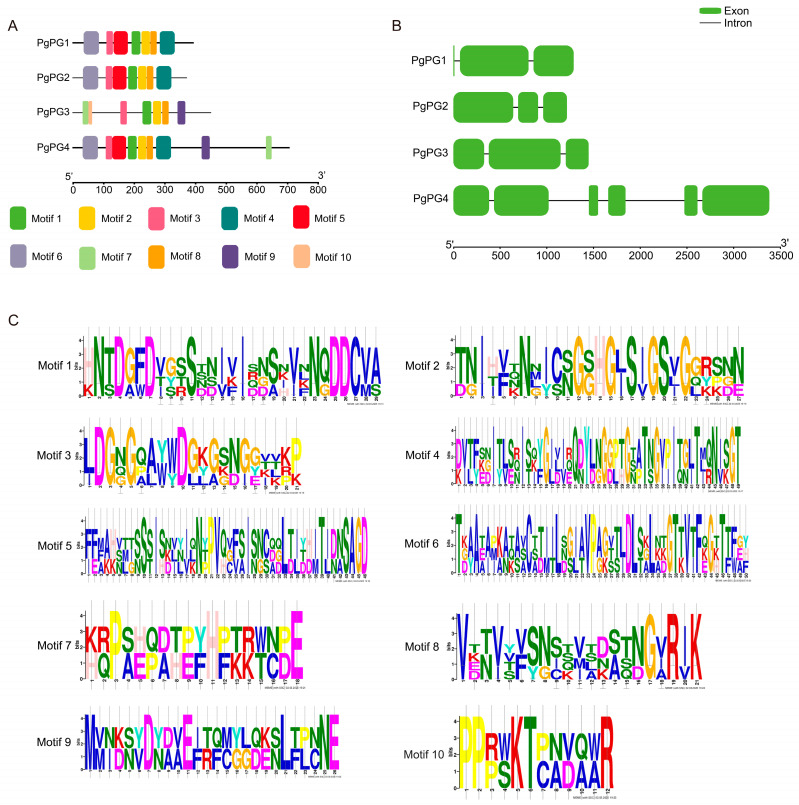
Conserved motif and gene structure analysis of PG family genes in *P. graminea*. (**A**) Distributions of conserved motifs in *PgPG* genes. Each motif is represented by a particular color. (**B**) The exon/intron structure of the *PgPG* genes. Green represents the exon regions, and the black lines represent introns. (**C**) The pattern identification of ten conserved sequences. The letters represent the amino acid sequence, and the size of the letters indicates the degree of sequence conservation; larger letters signify higher conservation.

The MEME tool (v5.5.7), annotated with InterPro (v87.0), identified ten conserved motifs (Figure 1A), and the conserved amino acid sequences are listed in Table 2. Gene structure analysis showed variability in intron numbers, with *PgPG4* containing five introns, while the other three genes had two introns each (Figure 1B). Conserved amino acid residue analysis revealed that all *PgPG* proteins had at least one NTD, DD, GHG, and RIK: the NTD and DD were located in motif 1, and GHG and RIK were found in motif 2 and motif 8, respectively (Figure 1C, Table 2).

### 2.3. Phylogenetic Analysis of the PgPG Protein Family

**Figure 2:**
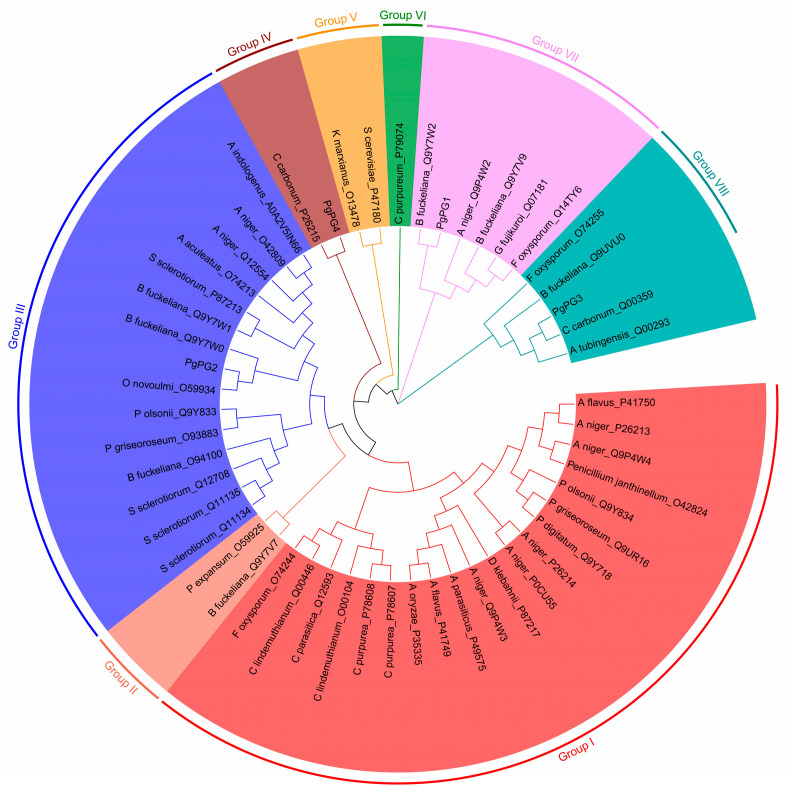
Circular cladogram showing the phylogenetic relationships of the 53 fungal PG proteins. The phylogenetic tree was generated using MEGA with the maximum likelihood method, employing 1000 bootstrap replicates. The same color indicates proteins with the same domain structure.

Phylogenetic analysis using the neighbor-joining method classified *PgPG* proteins into eight subfamilies, comprising 20, 2, 15, 2, 2, 1, 6, and 5 members each. *PgPG1* clustered with the PG gene of *Botryotinia fuckeliana*, indicating close evolutionary relatedness, while *PgPG4* shared a branch with *Cochliobolus carbonum* (Figure 2), revealing functional diversification within the *PgPG* protein family.

### 2.4. PgPG Gene Expression During Infection Stages

**Figure 3:**
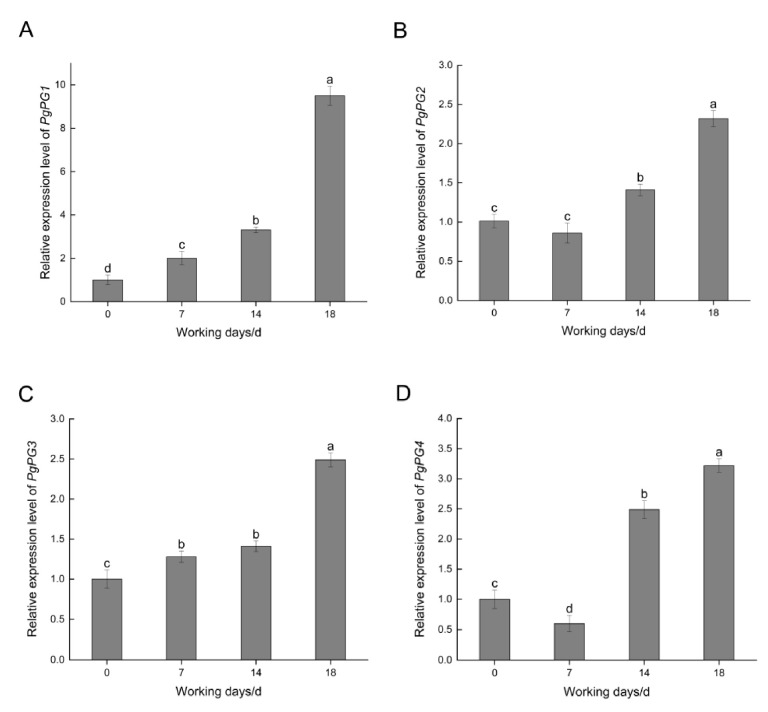
Expression level of *PgPG* genes in the interaction period between *P. graminea* and barley. (**A**) Expression level of *PgPG1* gene at different infection periods. (**B**) Expression level of *PgPG2* gene at different infection periods. (**C**) Expression level of *PgPG3* gene at different infection periods. (**D**) Expression level of *PgPG4* gene at different infection periods. Samples were collected at 0, 7, 14, and 18 d after inoculation. The expression level of each gene is compared to the abundance of *actin*, which was used as a reference gene. Different lowercase letters show standard errors based on three technical replicates.

All four *PgPG* genes were expressed during *P. graminea* infection of the barley cultivar Alexis. The expression levels of *PgPG1* and *PgPG3* increased consistently over time, while *PgPG2* and *PgPG4* slightly declined by 7 days post-infection before gradually increasing. All four *PgPG* genes reached the highest relative expression at 18 days post-infection (Figure 3). Among them, *PgPG1* exhibited the highest relative expression across all time points, warranting further investigation into its role in pathogenesis.

### 2.5. Signal Peptide Functionality, Capability of Inducion Cell Death, and Subcellular Location of PgPG1

**Figure 4:**
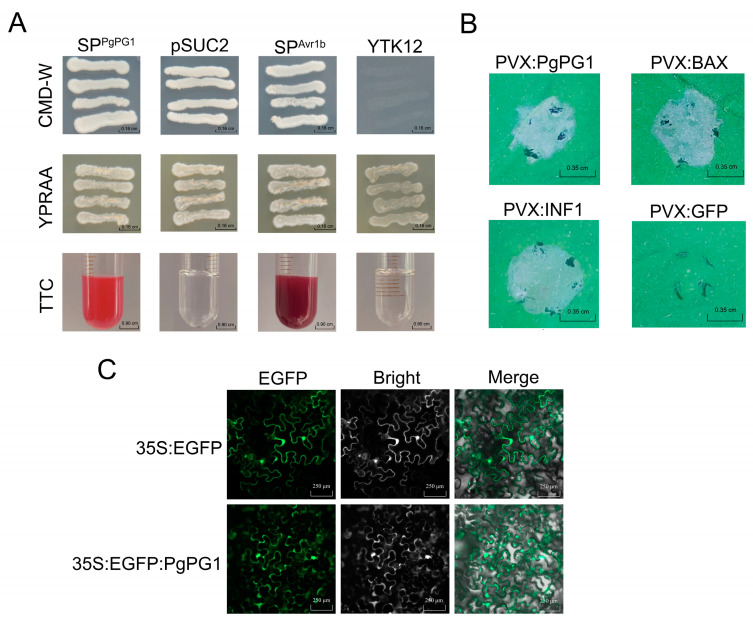
Functional verification of signal peptide, cell death detection and subcellular location of PgPG1. (**A**) The signal peptide (SP) functionality of PgPG1 was validated using a yeast signal trap assay. The yeast strain YTK12 was unable to grow on CMD-W medium (which lacks tryptophan) unless it contained the pSUC2 vector, which allows for the expression of the Trp operon. When the functional SP of PgPG1 is fused in-frame with the mature yeast invertase, it enables the secretion of invertase, allowing the yeast to grow on YPRAA medium. The known functional SP of Avr1b was utilized as a positive control. (**B**) The ability of PgPG1 from *P. graminea* to induce cell death was assessed in *N. benthamiana*. The leaves of 3-week-old *N. benthamiana* were infiltrated with *Agrobacterium tumefaciens* harboring the gene of interest. INF1 (BAX) and green fluorescent protein (GFP) were used as positive and negative controls, respectively. Photographs were taken 72 h after agroinfiltration. (**C**) Subcellular location of PgPG1 in *N. benthamiana*. PgPG1-EGFP fusion proteins or EGFP were expressed in *N. benthamiana*.

The TTC staining results showed that Avr1b and the *PgPG1* signal peptide facilitated the secretion of invertase, which degraded sucrose into monosaccharides. In contrast, neither the blank control YTK12 nor pSUC2 exhibited this activity (Figure 4A and Figure S2). These results confirm that the signal peptide from *PgPG1* has a functional secretory role. Transient expression of the *PgPG1* protein in *N. benthamiana* was performed using the potato virus X (PVX) expression system, with the results showing that *PgPG1* could induce cell death in *N. benthamiana* (Figure 4B).

To determine the subcellular localization of *PgPG1*, a fusion protein of *PgPG1* and GFP was transiently expressed in *N. benthamiana*. Confocal microscopy analysis revealed green fluorescence primarily localized to the cell membrane and nucleus, with minor fluorescence detected in the cytoplasm, suggesting that the *PgPG1* gene is expressed predominantly in the cell membrane and nucleus (Figure 4C).

### 2.6. Identification of PgPG1 Transformants

To confirm the presence of positive transformants, including RNAi transformants and expression transformants, we verified the presence of the hygromycin phosphotransferase gene, which acted as a reporter gene for screening transformants containing an exogenous target fragment. PCR amplification produced a band of approximately 1000 bp in transformants containing RNAi and overexpression constructs (Figure S3). Four RNAi transformants (*PgPG1-RNAi-1*, *PgPG1-RNAi-2*, *PgPG1-RNAi-3*, and *PgPG1-RNAi-4*) (Figure S3A) and four overexpression transformants (*PgPG1-OE-1*, *PgPG1-OE-2*, *PgPG1-OE-3*, and *PgPG1-OE-4*) (Figure S3B) were identified.

**Figure 5:**
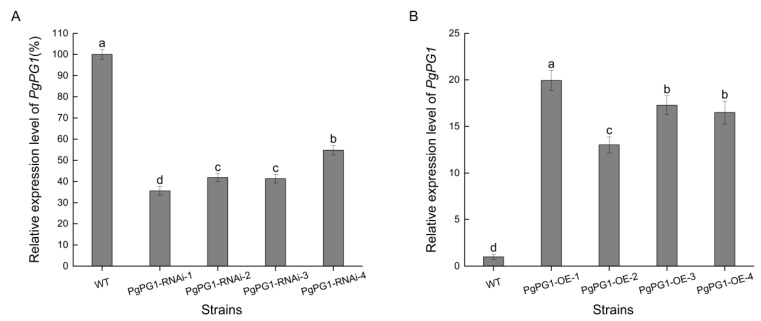
Relative expression of *PgPG1*-gene-transforming strains. (**A**) *PgPG1* relative expression of RNAi mutants. (**B**) *PgPG1* relative expression of overexpression mutants. Different lowercase letters show standard errors based on three technical replicates.

qRT-PCR analysis was performed on the transformants to assess *PgPG1* gene expression levels. Relative expression levels of *PgPG1-RNAi-1*, *PgPG1-RNAi-2*, *PgPG1-RNAi-3*, and *PgPG1-RNAi-4* were measured at 35.58%, 41.89%, 41.33%, and 54.86%, respectively, indicating decreases of 64.42%, 58.11%, 58.67%, and 45.14% compared to the wild-strain (Figure 5A). In contrast, relative expression levels of the overexpression transformants (*PgPG1-OE-1*, *PgPG1-OE-2*, *PgPG1-OE-3*, and *PgPG1-OE-4*) were significantly elevated, 19.94%, 13.03%, 17.28%, and 16.50% higher than the wild-strain, respectively (Figure 5B).

### 2.7. Role of PgPG1 in Vegetative Growth and Polygalacturonase Activity

**Figure 6:**
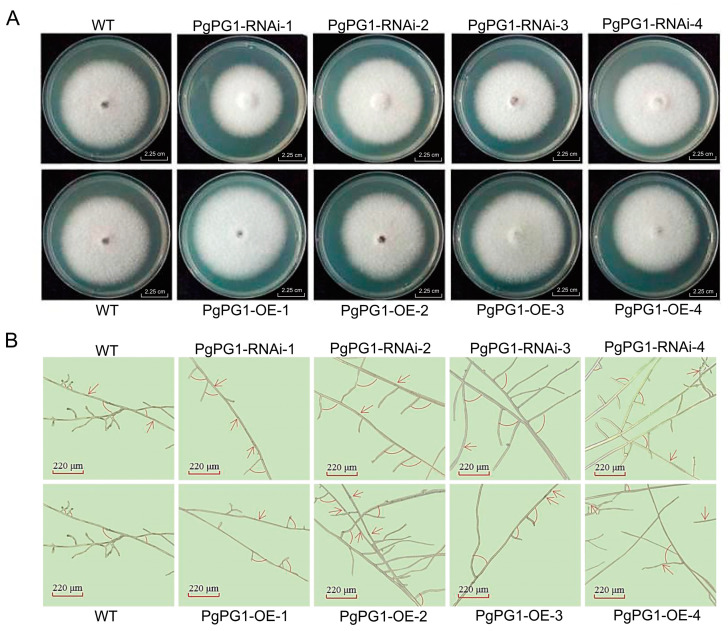
WT and mutant strains of *P. graminea* grown in PDA medium. (**A**) Mycelia growth of the WT and *PgPG1* mutants on PDA for 7 days. (**B**) Microscopic structural examination of *PgPG1* mutants and WT incubated on PDA for 7 days. The red arrow indicates the position of mycelium distortion.

The colony morphology of wild-strain and *PgPG1* mutants was observed after 7 days of growth on PDA. All strains exhibited similar morphology, characterized as white, flat, relatively compact, and villose (Figure 6A), indicating that the *PgPG1* gene does not influence colony morphology. Measurements of colony diameter revealed that all *PgPG1* RNAi mutants had smaller diameters than the wild-strain (Figure 6A). Among the overexpression mutants, *PgPG1-OE-2* had a smaller diameter, while *PgPG1-OE-1* and *PgPG1-OE-3* had larger diameters than the wild-strain. The diameter of *PgPG1-OE-4* was comparable to the wild-strain. These findings suggest that the *PgPG1* gene plays a role in *P. graminea* growth.

**Figure 7:**
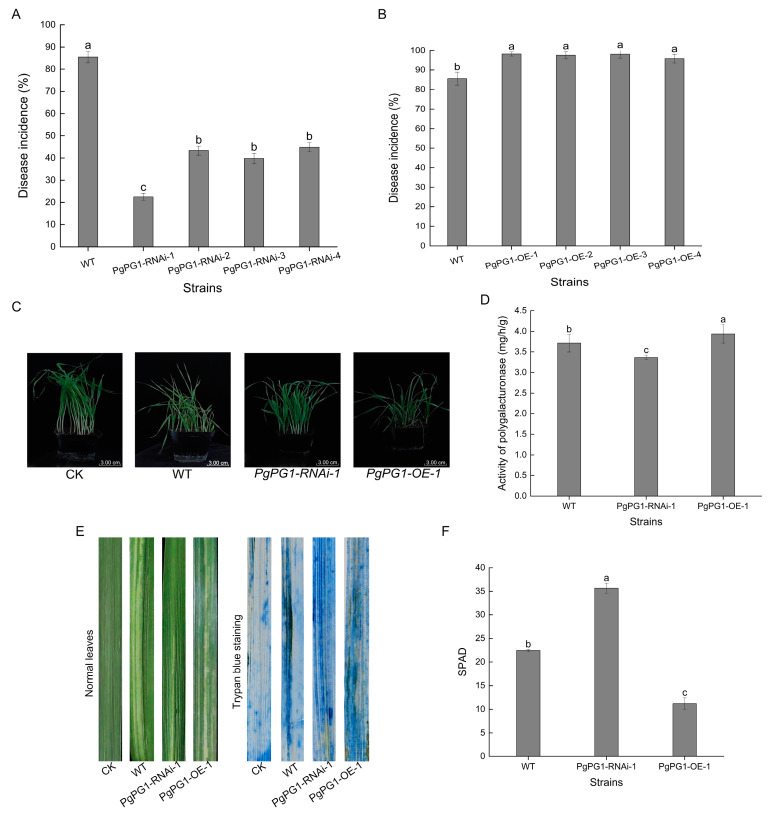
Pathogenicity detection of *PgPG1* mutant strains of *P. graminea*. (**A**,**B**) Disease incidence of barley that had been inoculated with WT and mutant strains for 20 days at 18 °C. (**C**) Growth trends of barley seeds inoculated with fungal mycelium from WT and transformants for 20 days at 4 °C. Barleys were observed after sowing for 14 days. The CK is Alexis plants uninfected by *P. graminea*. (**D**) Activity evaluation of the secreted polygalacturonase of WT, *PgPG1-RNAi-1*, and *PgPG1-OE-1*. (**E**) Trypan blue staining to detect the degree of cell necrosis in WT, *PgPG1-RNAi-1*, and *PgPG1-OE-1* leaves. The CK is Alexis leaves uninfected by *P. graminea*. (**F**) The content of SPAD in Alexis infected by WT, *PgPG1-RNAi-1*, and *PgPG1-OE-1*, respectively. Different letters indicate a significant difference between the WT and transformants (*p* < 0.05). Bars denote standard errors from three repeated experiments.

Polygalacturonase activity was measured in the *PgPG1* mutant to assess the role of the *PgPG1* gene in PG activity. The results showed that *PgPG1-OE-1* had 6.11% higher PG activity than the wild-type, while *PgPG1-RNAi-1* had 9.38% lower PG activity (Figure 7D).

### 2.8. Impact of PgPG1 on Pathogenicity

The disease incidence of the barley cultivar Alexis was recorded for the infection of RNAi and overexpression mutants. Infections with RNAi mutants *PgPG1-RNAi-1*, *PgPG1-RNAi-2*, *PgPG1-RNAi-3*, and *PgPG1-RNAi-4* resulted in disease incidences of 22.58%, 43.33%, 39.78%, and 44.85%, respectively (Figure 7A). Infections with overexpression mutants *PgPG1-OE-1*, *PgPG1-OE-2*, *PgPG1-OE-3*, and *PgPG1-OE-4* led to disease incidences of 98.25%, 97.62%, 98.15%, and 95.83%, respectively (Figure 7B). Relative to the wild-type, disease incidence decreased by 62.94%, 42.19%, 45.74%, and 40.67% for RNAi mutants, and increased by 12.73%, 12.10%, 12.63%, and 10.31% for overexpression mutants. These findings indicate that the *PgPG1* gene plays a significant role in the pathogenicity of *P. graminea*.

The growth of plants uninfected by *P. graminea* and those infected by different strains were observed. Because these plants were not infected by *P. graminea*, the growth of the plant in CK (Alexis plants uninfected by *P. graminea*) is the best in Figure 7C; plants infected with *PgPG1-RNAi-1* performed the second best, and plants infected with *PgPG1-OE-1* performed the worst (Figure 7C). To further assess tissue damage, trypan blue dye was used to stain entire barley leaves infected with the wild-type, *PgPG1-RNAi-1*, and *PgPG1-OE-1* strains. Following staining and decolorizing with chloral hydrate, leaves infected with *PgPG1-OE-1* showed greater damage than the control group. In contrast, leaves infected with *PgPG1-RNAi-1* exhibited minimal damage, and no staining was observed in the leaves of the control group (Figure 7E). We examined the chlorophyll content of the leaves infected by the wild-type, *PgPG1-RNAi-1*, and *PgPG1-OE-1* strains, and the SPAD value of leaves infected by *PgPG1-RNAi-1* was the highest; however, that of leaves infected by *PgPG1-OE-1* was the lowest (Figure 7F).

## 3. Discussion

Fungal pathogens interact with host plants by degrading cell walls, often using PG enzymes as the initial tools for breaking down these structures [21]. We identified and characterized the *PgPG1* gene from *P. graminea*, evaluating its role in growth, infection, and pathogenicity. This work provides insights into the molecular mechanisms underlying fungal pathogenicity, emphasizing the importance of PG genes in host–pathogen interactions.

Polygalacturonase genes play varied roles in fungal pathogenicity. For example, necrotrophic fungi often possess more *GH28* genes than non-pathogenic or biotrophic fungi [22]. *Phytophthora cinnamomi* contains a large *endoPG* gene family with 19 members [23]. In *P. graminea*, we identified four *PgPG* genes at the whole-genome level, demonstrating extensive diversification through differences in intron–exon structures.

Expression analysis during the infection stages revealed that *PgPG1* and *PgPG3* were upregulated over time, with *PgPG1* exhibiting the highest relative increase. This pattern suggests that *PgPG1* is integral to degrading polygalacturonic acid, a primary component of plant cell middle lamellae. These findings identify *PgPG1* as a key player in *P. graminea* pathogenicity.

Fungal *PG* genes exhibit functional differentiation in their roles in pathogenicity. Some studies have demonstrated that certain *PG* genes contribute significantly to fungal virulence, including *pecA* gene in *Aspergillus flavus* [24], *BcPG1* in *Botrytis cinerea* [25], *PG1* in *Fusarium oxysporum* [14], *CpPG1* and *CpPG2* in *Claviceps purpurea* [16], the *PGLA* gene in *Xylella fastidiosa* [17], and many others. Conversely, other studies have shown that some *PG* genes do not contribute to pathogenicity, including *enPG1* in *Cryphonectria parasitica* [18], *PG5* in *F. oxysporum* [19], and *AaPG1* in *A. alternat* [15]. This apparent discrepancy may stem from the functional redundancy of *PG* genes. These findings highlight the controversial role of the *PG* gene in host–pathogen interactions.

Cell-wall-degrading enzymes serve as tools for pathogenic fungi to breach host defenses and as elicitors that trigger plant immune responses, acting as critical warning signals [26]. For instance, PG from *Sclerotinia sclerotiorum* can induce plant cell death [27]. Secreted effectors from diverse organisms have been extensively studied and linked to various physiological functions, including cell proliferation, immune responses, and pathogenic mechanisms [28,29]. To fully understand PG’s role in plant–pathogen interactions, it is essential to consider its pathogenic function and capacity to induce immune responses. In *B. cinerea*, at least five PGs have been confirmed to trigger immune responses in host plants [30]. Similarly, proteins like VmE02 from *Valsa mali* and cerato-platanin SsCP1 from *Sclerotinia sclerotiorum* can induce cell death independently of signal peptide presence [31]. This study identified that *PgPG1* possesses secretory activity and induces cell death. Moreover, subcellular localization studies revealed *PgPG1*’s presence in the cell membrane and nucleus, where maybe the interaction regions between PgPG1 and the host cause an immune response. These findings underscore the importance of the apoplastic space in *PgPG1*’s functional activity, providing new insights into its contribution to *P. graminea* pathogenicity.

The pathogenic process of fungal infection in host plants is complex, with successful invasion and establishment within the host being critical for the pathogen’s impact [32,33]. Polygalacturonase, secreted by pathogenic fungi, plays a pivotal role by degrading the middle lamella and the intercellular layers of the plant’s primary cell wall, facilitating the pathogen’s smooth infiltration [34]. Research has consistently shown the strong association between *PG*s and fungal pathogenicity. For instance, mutating the *PG* gene in *Alternaria citri* significantly reduces the incidence of citrus and horse boll infections [15]. Similarly, the loss of *BcPG1* in *Botrytis cinerea* markedly decreases pathogenicity across various host plants [35]. In *Claviceps purpurea*, deletion of the *endo-PG* gene results in near-complete loss of pathogenicity to rye [16]. In this study, we explored the role of *PgPG1* in *P. graminea* by generating RNAi and overexpression mutants, which revealed its essential function in infection-related morphogenesis. Our results demonstrated that the *PgPG1-RNAi* mutants exhibited reduced pathogenicity, while the *PgPG1-OE* mutants displayed enhanced pathogenicity in barley. These findings were corroborated by trypan blue staining and polygalacturonase activity assays, which confirmed *PgPG1*’s involvement in facilitating pathogenicity.

In conclusion, we identified *PgPG1* as a key pathogenicity factor in *P. graminea*. Our study highlights its critical roles in fungal growth, host infection, and disease development, advancing our understanding of the mechanisms underlying fungal pathogenicity.

## 4. Materials and Methods

### 4.1. Plant Materials, Strains, and Culture Conditions

Barley cultivar Alexis was used for inoculation assays. The *P. graminea* strain QWC (wild-type) was cultured at 25 °C on PDA medium, comprising 200 g/L potato decoction, 20 g/L D-glucose anhydrous, and 17 g/L agar.

### 4.2. Identification of PG Genes from the P. graminea Genome

Genomic data of *P. graminea* were downloaded from NCBI (BioProject: PRJNA373892). The Hidden Markov Model corresponding to the peptidase Glyco_hydro_28 domain (PF00295) was obtained from the Pfam database. Candidate *PG* genes were identified using HMMER 3.3.2 [36] with an E-value threshold <e^−10^, and their Glyco_hydro_28 conserved domain was confirmed using CDD research [37].

### 4.3. Sequence Feature, Gene Structure, and Motif Analysis

Protein-coding sequences (CDSs) and amino acid sequences of PG were extracted from the *P. graminea* genome. ProtParam (https://web.expasy.org/protparam/, accessed on 14 September 2021) [38] was used to calculate properties such as amino acid count, molecular weight, isoelectric point (pI), grand average of hydropathicity (GRAVY) index, instability index, and aliphatic index of PgPG proteins. SignalP6.0 [39] and Cell-PLoc (v2.0) [40] were used to predict signal peptides and subcellular locations. Exon–intron positions were mapped with TBtools (v2.121) [41], while conserved motifs were analyzed using MEME with default parameters (motif set to 10) and visualized using TBtools.

### 4.4. Phylogenetic Tree Construction

Protein sequences with locus IDs were retrieved from NCBI to perform a phylogenetic analysis of PgPG and other pathogenic fungi. Phylogenetic trees were constructed using MEGA 7 with the maximum likelihood and neighbor-joining methods, and graphical representations were refined using the Evolview website.

### 4.5. RNA Extraction and Gene Expression Assay

For gene expression analysis, sterilized barley seeds (cultivar Alexis) were inoculated by sandwiching them between two layers of *P. graminea* mycelia grown for seven days and then incubating at 6 °C. Infected seed embryos were collected at 7, 14, and 18 d post-infection and rapidly frozen in liquid nitrogen. Mycelia pre-infection were used as the control. RNA was extracted using a TRIzol RNA extraction kit, and cDNA was synthesized using a FastKing One Step RT-qPCR kit (Tiangen, Beijing, China, FP313). Relative expression levels were calculated using the 2^−∆∆Ct^ method. All primers used are listed in Table 3, and qPCR analysis was performed with three independent biological replicates.

### 4.6. Plasmid Construction

Primers for the RNAi segments, including positive and reverse segments, to amplify the *PgPG1* gene were created using the E-RNAi (v3.2) based on the CDS region of the *PgPG1* gene, including responsive restriction sites. The positive segment included *Kpn* I and *Xho* I sites, and the reverse segment included *Bgl* II and *Hind* III sites (Table 3; Figure S1). The amplification process comprised 1 μL cDNA template, 1 μL each of forward and reverse primers, 12.5 μL Premix Taq, and 9.5 μL ddH_2_O. The RNAi segments were cloned following the protocol for the *PgPG1* gene clone. The RNAi segments and pSilent-1 vector were digested using *Kpn* I/*Xho* I and *Bgl* II/*Hind* III, respectively. Initially, the forward RNAi segment was ligated into pSilent-1 using T4 ligase, and then the reverse RNAi segment was ligated into the pSilent-1 vector containing the forward RNAi segment, resulting in the construction of RNAi pSilent-1: *PgPG1*. For the *PgPG1* gene clone, the fragment was digested with *Xho* I and *Eco*R I (Table 3; Figure S1) and subsequently ligated into the pBARGPE1-Hyg vector, resulting in the construction of the overexpression plasmid pBARGPE1-Hyg: *PgPG1*.

### 4.7. Transient Expression of PgPG1 in Nicotiana Benthamiana

The PVX: *PgPG1* construct was transformed into *A. tumefaciens* GV3101 and cultured in LB medium at 28 °C at 200 rpm for 48 h. The bacterial pellets were collected after centrifugation at 3500 rpm for 5 min, resuspended in the infiltration buffer (10 mM MgCl_2_, 10 mM MES, 100 μM AS; pH = 5.7), centrifuged again at 3500 rpm for 5 min, and adjusted to OD_600_ = 0.4. The *Agrobacterium* suspension was infiltrated into the abaxial side of 3-week-old *N. benthamiana* leaves using a 1 mL syringe without a needle. Plants were grown under 16 h light/8 h dark conditions at 24 °C, and symptoms were observed 3–5 days post-infiltration.

### 4.8. Signal Peptide Functional Validation

Primers for *PgPG1* PCR amplification, including gene clones and subcellular locations, were designed using DNAMAN (v5.2.2). After PCR amplification, the target fragment of the *PgPG1* gene was recovered using a DNA recovery kit (Tiangen, Beijing, China, DP204), ligated to a T-vector, and transformed into *E. coli* DH5α. Following blue–white selection, positive clones were identified, and the *E. coli* cultures containing these clones were sent to a professional sequencing company (Tsing ke, Xi’an, China) for sequencing.

The yeast signal sequence trap system was used to verify the secretion function of the PgPG1 signal peptide. The YTK12 strain, a sucrase-deficient mutant, was used for functional validation. The chemically synthesized *PgPG1* signal peptide sequence was cloned into the pSUC2 vector, resulting in the pSUC2: *PgPG1*^sp^ construct (Table 3; Figure S1). This construction, the negative control vector pSUC2, and the positive control vector pSUC2-Avr1b were transformed into YTK12, respectively. If the PgPG1 signal peptide exhibited secretory functionality, the sucrase from YTK12 would be secreted successfully outside the cell. The hydrolysis of sucrose into monosaccharides by the secreted sucrase would then react with 2, 3, 5-triphenyl tetrazolium chloride (TTC) to produce a red triphenyl tetrazolium chloride precipitate, confirming the peptide’s secretion capability.

### 4.9. Subcellular Localization

The *PgPG1* CDS was inserted into the pCAMBIA1300-35S-EGFP vector (Table 3; Figure S1). This recombinant vector (pCAMBIA1300-35S-EGFP: *PgPG1*) was transformed into *N. benthamiana* leaves using an *Agrobacterium*-mediated transformation method. The leaves of 3–4-week-old tobacco plants were infiltrated and subsequently incubated in a controlled chamber at 22 °C with a 12 h light/dark cycle for 48–72 h. The distribution of green fluorescence in the transformed tobacco leaves was observed and photographed using a laser confocal scanning microscope with an excitation wavelength of 488 nm.

### 4.10. Protoplast Transformation

Protoplast preparation and genetic transformation were mainly referenced to Aragona and Porta-Puglia [42]. Mycelia cultured for 48 h were harvested by filtration through a sterilized funnel, washed three times, and blotted dry using sterile filter paper. Approximately 0.1 g of mycelia was added to 10 mL enzymatic hydrolysate in a wide-mouth conical flask and incubated at 30 °C with shaking at 80 rpm for 3 h. The enzymatic hydrolysis mixture was filtered using sterilized cellophane, and the filtrate was centrifuged at 1996 rpm for 5 min, discarding the supernatant. Protoplasts were obtained by washing twice with 300 μL of 1.2 mol/L NaCl and resuspended in 100 μL NTC for transformation.

pSilent-1: *PgPG1* DNA (1–10 μg) was added to the 100 μL protoplast suspension, blended, and incubated on ice for 20 min. Sequential additions of 200 μL, 200 μL, and 800 μL PTC were made, mixing well after each addition. Following a 15 min rest at room temperature, 3 mL rPD medium was added and gently mixed. The culture was incubated at 25 °C for 24 h and subsequently mixed with 20 mL rPDA medium before being incubated at 25 °C.

### 4.11. Selection and Identification of Mutants

Once mycelia appeared on PDA plates, the plates were overlaid with 10 mL PDA medium containing Hygromycin B. Single colonies were picked and subcultured on fresh PDA containing Hygromycin B for three consecutive cycles, resulting in the isolation of transformants. Positive transformants for RNAi and overexpression were identified through Hygromycin phosphotransferase gene segment amplification and qRT-PCR using the primers listed in Table 3.

### 4.12. Morphology Observation and Transformant Colony Diameter Measurement

Agar disks (5 mm diameter) containing mycelia were placed on blank PDA plates and incubated in the dark at 25 °C. Colony morphology was observed and photographed, and colony diameters were measured. Three biological replicates were used for each strain.

After placing the mycelia in the center of the PDA plate, a coverslip was inserted into the PDA at a 45° angle, 1 cm away from the mycelia. Mycelium morphology was observed under an optical microscope when the mycelia covered two-thirds of the coverslip.

### 4.13. Pathogenicity Testing of Mutants

The highly susceptible barley cultivar Alexis was used to test the pathogenicity of the transformants. Surface-sterilized Alexis seeds were sandwiched between PDA plates covered with 7-day-old mycelia and incubated in the dark at 25 °C for 20 days. The thirty infected seeds were then planted in pots containing a mixture of vermiculite and nutrient soil. The plants were grown under controlled conditions in a phytotron with alternating 12 h of light at 16 °C and 12 h of dark at 12 °C cycles. When stripe symptoms appeared, diseased plants were counted, and disease incidence was calculated for three biological replicates.

### 4.14. Trypan Blue Staining

Leaves infected for three weeks were harvested, rinsed with water, and stained with 0.4% (*w*/*v*) trypan blue solution for 3 min in boiling water. The staining solution was then removed before applying a decolorizing solution. Leaves were decolorized for 48 h until they appeared transparent.

### 4.15. Determination of PG Activity

Wild-type and mutant strains were cultured on PDA medium at 25 °C for seven days. The mycelia were then harvested for PG activity analysis, following the protocol outlined in the polygalacturonase test kit (Aoqing, Nanjing, China, SP7096).

## 5. Conclusions

In this study, we identified four PgPG genes (PgPG1-PgPG4) in the *P. graminea* genome and obtained the highest inducible expression of the *PgPG1* gene during barley infection. In addition, the PgPG1 was verified to be a secretory protein and had the ability to cause immune response. The *PgPG1* gene was involved in the pathogenicity of *P. graminea* in barley and has great potential as a pathogen target gene to develop a durable resistance variety to *P. graminea*.

## Figures and Tables

**Table 1:** Physical and chemical properties of PG genes of *P. graminea*.

Gene	Gene ID	Number of Amino Acids/aa	Molecular Weight/kD	Isoelectric Point	GRAVY Index	Instability Index	Aliphatic Index	Signal Peptide Length	Subcellular Location
*PgPG1*	P.graminea_GLEAN_10002343	392	41.62	5.60	−0.174	31.85	81.10	18	extracellular
*PgPG2*	P.graminea_GLEAN_10002483	370	37.04	8.31	0.114	21.89	81.73	19	extracellular
*PgPG3*	P.graminea_GLEAN_10005830	448	48.31	6.24	−0.276	37.10	79.82	19	extracellular
*PgPG4*	P.graminea_GLEAN_10006636	705	74.98	5.98	−0.252	37.08	74.89	19	extracellular

**Table 2:** Conserved motifs of amino acids of *PgPG* genes family in *P. graminea*.

Conserved Motifs	E-Value	Number of Amino Acid	Amino Acid Sequence
Motif1	9.8 × 10^−13^	29	HNTDGFDVGSSSNIVIQNSNVLNQDDCVA
Motif2	2.2 × 10^−7^	27	TNIHFKNLICNGGHGJSIGSVGGRSNN
Motif3	1.5 × 10^−4^	21	LDGNGQAWWDGKGSNGGVVKP
Motif4	1.1 × 10^−4^	49	DVTFEDITLSRISKYGIVIQQDYLNGGPTGSATNGVPITGLTJQNISGT
Motif5	3.1 × 10^−4^	46	FFFAHNLISSSISDIYIQNPPVQVFSISNCDGLTIDHITIDNSAGD
Motif6	1.1 × 10^−3^	50	TGAAAAIKAKAACSTIILBGIAVPAGTTLDLSGLKDGTTVTFQGKTTFGY
Motif7	2.8 × 10^0^	18	KRPAEPDHPFHFKKWCDE
Motif8	2.1 × 10^1^	21	VETVTFSNSSVSDATNGVRIK
Motif9	1.5 × 10^1^	26	MMNDNVDNDVEFRFCGGDENLFPCNE
Motif10	5.4 × 10^1^	12	PPRWKTCNDQWR

**Table 3:** Primers information used in the study.

Code	Primer	Sequence (5′–3′)	Restriction Site	Purpose
1	*PgPG1*^SP^-*Eco*R I-F	CCGGAATTCATGCGTTCTAACGGGATTTT	*Eco*R I	Clone Pgpg1 to pSUC2 for signal peptide functional validation
2	*PgPG1*^SP^-*Xho* I-R	CCGCTCGAGGGCCTGCCCTACGGCGGCAA	*Xho* I	
3	*PgPG1*-SL-*Bam*H I-F	CGGGATCCATGCGTTCTAACGGGATTTT	*Bam*H I	Clone Pgpg1 to EGFP for subcellular localization assay in *N. benthamiana*
4	*PgPG1*-SL-*Sma* I-R	TCCCCCGGGCGCTGCTAGCTTGGAAACTG	*Sma* I	
5	*PgPG1*-RNAi-*Kpn* I-*Xho* I-F	GGGGTACCCCGCTCGAGGAGCAGCACCAACATCAAGA	*Kpn* I-*Xho* I	Clone RNAi segment to pSilent-1 for the construction of pSilent-1: *Pgpg1*
6	*PgPG1*-RNAi-*Bgl* II-*Hind* III-R	GAAGATCTCCCAAGCTTCCACCGCCAGTAATATCCAC	*Bgl* II-*Hind* III	
7	*PgPG1*-OE-*Eco*R I-F	GGAATTCATGCGTTCTAACGGGATTTT	*Eco*R I	Clone *Pgpg1* to pBARGPE1-Hyg-mCherryfor the construction of pBARGPE1-Hyg: *Pgpg1*
8	*PgPG1*-OE-*Xho* I-R	CCGCTCGAGCGCTGCTAGCTTGGAAACTG	*Xho* I	
9	*Hyg B*-F	ATGAAAAAGCCTGAACTCAC		Generation of *Pgpg1* mutants by the amplificationg segment of hygromycin resistance gene in *Pyrenophora graminea*
10	*Hyg B*-R	CTATTCCTTTGCCCTCGGA		
11	*PgPG1*-RNAi-RT-F	TCAAGATCCGCGACAGCAT		Expression level analysis of *Pgpg1* genes in RNAi mutant of *Pyrenophora graminea*
12	*PgPG1*-RNAi-RT-R	TGGCTGCCGTTGGAGTACA		
13	*PgPG1*-OE-RT-F	CTGGACATTTACCACATCACTCTGA		Expression level analysis of *Pgpg1* genes in OE mutants of *Pyrenophora graminea*
14	*PgPG1*-OE-RT-R	CGTCAAATCCGTCGCTGTTA		
15	*Actin*-F	GCGGTTACACCTCTCTACCAC		Expression level analysis of internal control gene *Actin* in *Pyrenophora graminea*
16	*Actin*-R	AGTCTGGATCTCCTGCTCAAAG		

## Data Availability

Data can be provided by the corresponding author upon reasonable request.

## Supplementary Materials

The following supporting information can be downloaded at: https://www.mdpi.com/article/10.3390/ijms26062401/s1.
