# Supplementary material for: The Essentials of PgPG1, a Polygalacturonase-Encoding Gene for the Invasion of Pyrenophora graminea to Hordeum vulgare
Source: Int J Mol Sci. 2025 Mar 7;26(6):2401. doi: 10.3390/ijms26062401 (PMC11942426; doi:10.3390/ijms26062401)
Supplement: Supplementary file 1 [file ijms-26-02401-s001.zip › ijms-3357632-supplementary.pdf]

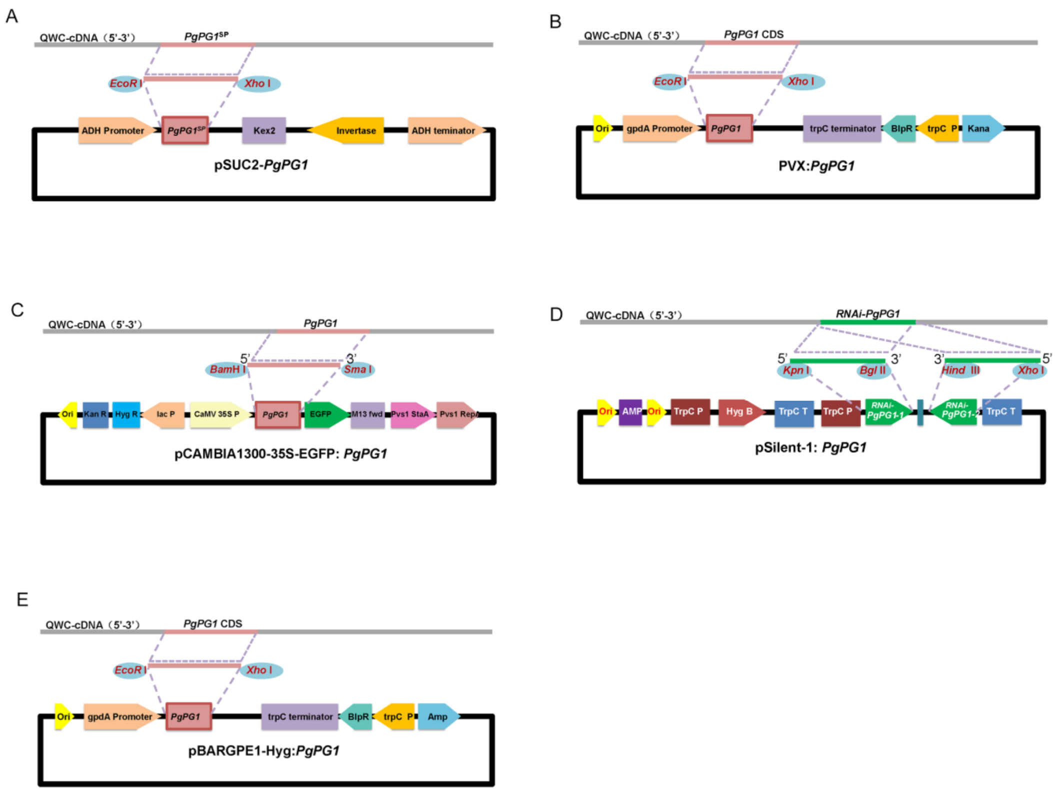

Figure S1. Schematic diagram of *PgPG1* gene vector construction. (**A**)Signal peptide secretion system of *PgPG1* gene. (**B**)Transient expression vector of *PgPG1* gene. (**C**)Subcellular localization vector of *PgPG1* gene. (**D**) Interference vector of *PgPG1* gene. (**E**) Overexpression vector of *PgPG1* gene.

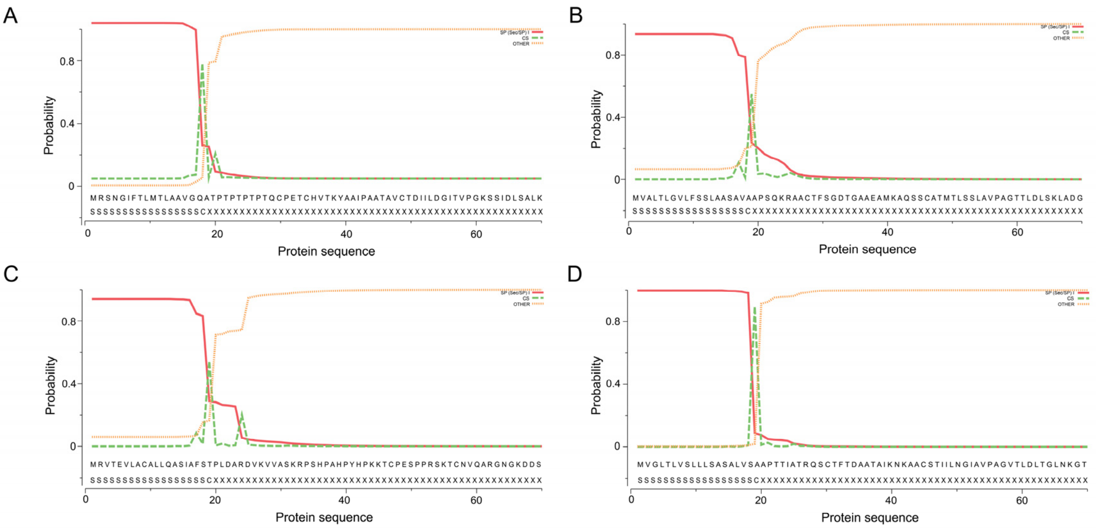

Figure S2. Prediction of signal peptide sequence of PgPGs in *P. graminea*. (**A**) Prediction of signal peptide sequence of PgPG1. (**B**) Prediction of signal peptide sequence of PgPG2. (**C**) Prediction of signal peptide sequence of PgPG3. (**D**) Prediction of signal peptide sequence of PgPG4.

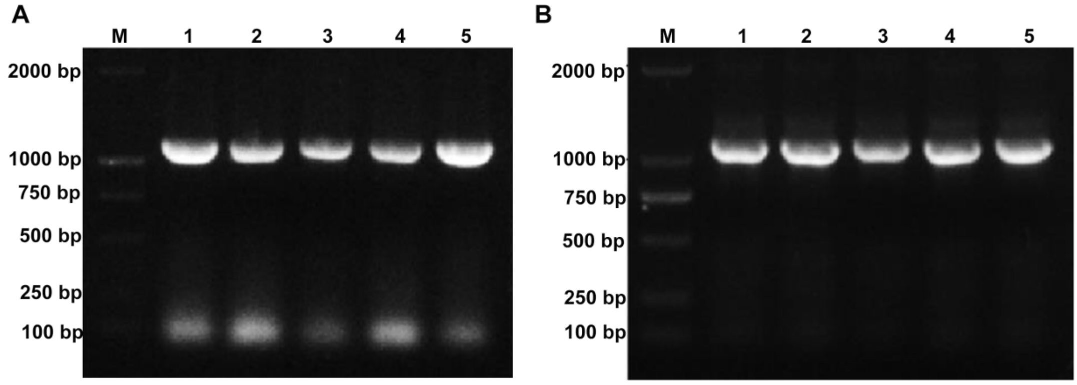

Figure S3. Hygromycin-resistant PCR validation of *PgPG1* gene-transforming strains. (**A**) M is DL 2000 DNA marker; Lane 1-4 are the *Hyg* gene bands amplified by the single colony of pSilent-1:*PgPG1* recombinant vector transformed by different *P. graminea*, and lane 5 is the *Hyg* gene band amplified by pSilent-1 empty vector; (**B**) M is DL 2000 DNA marker; Lane 1-4 are the *Hyg* gene bands amplified by single colony of pBARGPE1-Hyg:*PgPG1* recombinant vector transformed by different *P. graminea*, and lane 5 is the *Hyg* gene band amplified by pBARGPE1-Hyg empty vector.
